# Utilization of radiation therapy and predictors of noncompliance among Syrian refugees in Turkey

**DOI:** 10.1186/s12885-022-09558-5

**Published:** 2022-05-12

**Authors:** Mutlay Sayan, Mehmet Fuat Eren, Sarah Sabrine Kilic, Ayse Kotek, Sedenay Oskeroglu Kaplan, Ozge Duran, Funda Cukurcayır, Ibrahim Babalıoglu, Ozlem Derinalp Or, Gul Aysen Ozturk, Celalettin Eroglu, Fatma Teke, Neslihan Kurtul, Tugce Kutuk, Beyhan Ceylaner Bicakci, Sukran Senyurek, Meryem Aktan, Swati Mamidanna, Nisha Ohri, Bruce Haffty, Banu Atalar

**Affiliations:** 1 Department of Radiation Oncology, Dana-Farber Cancer Institute, Brigham and Women’s Hospital, Harvard Medical School, 75 Francis Street. ASB1 - L2, Boston, MA 02115 USA; 2 Marmara University Pendik Education and Research Hospital, Istanbul, Turkey; 3 Taussig Cancer Institute, Cleveland Clinic Foundation, Cleveland, OH USA; 4 Gaziantep Dr. Ersin Arslan Education and Research Hospital, Gaziantep, Turkey; 5 Sanliurfa Mehmet Akif Inan Education and Research Hospital, Sanliurfa, Turkey; 6 Balikesir Ataturk City Hospital, Balikesir, Turkey; 7 Konya Education and Research Hospital, Konya, Turkey; 8 Adana City Education and Research Hospital, Adana, Turkey; 9 Bursa Ali Osman Sonmez Oncology Hospital, Bursa, Turkey; 10 Erciyes University, Kayseri, Turkey; 11 Dicle University, Diyarbakir, Turkey; 12 Kahramanmaras Sutcu Imam University, Kahramanmaras, Turkey; 13 Malatya Education and Research Hospital, Malatya, Turkey; 14 Dr. Lutfi Kirdar Kartal Education and Research Hospital, Istanbul, Turkey; 15 Kahramanmaras Necip Fazil City Hospital, Kahramanmaras, Turkey; 16 Necmettin Erbakan University, Konya, Turkey; 17 Rutgers Cancer Institute of New Jersey, New Brunswick, NJ USA; 18 Acibadem Maslak Hospital, Istanbul, Turkey

## Abstract

**Background:**

Access to cancer care is a problem that continues to plague refugees displaced from their home countries. The turbulent political crisis in Syria, which has led to millions of refugees seeking asylum in Turkey, merits further attention. We aimed to study the rate of utilization of radiation therapy among Syrian refugees with cancer living in Turkey in an attempt to identify the contributing factors predictive of non-compliance with prescribed RT.

**Methods:**

In this retrospective review of 14 institutional databases, Syrian refugee patients in Turkey with a cancer diagnosis from January 2015 to December 2019 who were treated with RT were identified. The demographic data, treatment compliance rates, and toxicity outcomes in these patients were surveyed. Variable predictors of noncompliance such as age, sex, diagnosis, treatment length, and toxicity were studied. The association between these variables and patient noncompliance was determined.

**Results:**

We identified 10,537 patients who were diagnosed with cancer during the study period, of whom 1010 (9.6%) patients were treated with RT. Breast cancer (30%) and lung cancer (14%) were the most common diagnoses with up to 68% of patients diagnosed at an advanced stage (Stage III, IV). 20% of the patients were deemed noncompliant. Treatment with concurrent chemoradiotherapy (OR 1.61, 95% CI 1.06–2.46, *p* = 0.023) and living in a refugee camp (OR 3.62, 95% CI 2.43–5.19, *p* < 0.001) were associated with noncompliance. Age, sex and treatment length were not significantly associated with noncompliance.

**Conclusions:**

Noncompliance with radiotherapy among Syrian refugees in Turkey remains an area of concern with a multitude of factors contributing to these alarming numbers. Further studies to better ascertain the finer nuances of this intricately complex problem and a global combination of efforts can pave the way to providing a solution.

## Background

As political unrest continues to rage in the Middle East, Syria is still in a state of an ongoing, turbulent Civil War since its onset in 2011. After years of fighting against unjust oppression, millions of refugees have fled from the country to escape from the harsh circumstances that have been inflicted upon them, in search of a better way of life. Of the approximately 5.6 million people who have been displaced to the neighboring countries, Turkey is currently hosting the largest number of registered refugees, far more than any other country [1]. Sharing a large common land border with Syria, making it accessible to this new population influx, Turkey has stepped forward during this critical time to provide shelter to almost 3.6 million of these troubled people, with the numbers only increasing with time.

This inadvertent migration allowed for an escape from the war but has however presented the refugees with a different set of challenges as they struggle to adapt to their new homes. One of the major concerns has been the influence on healthcare, particularly cancer care. Access to health care is enshrined as a human right in the Turkish Constitution and staying true to this principle, in April 2013, Turkey has passed its first law on asylum—the “Law on Foreigners and International Protection”. This new set of regulations authorized the provision of free medical treatment to registered refugees in the manner generally provided to Turkish citizens, including cancer treatment and care at tertiary government and university hospitals [2–4].

Despite these amendments to facilitate the migrant population, an inconsistency between the healthcare services provided and services availed has been observed, primarily arising from the fact that this population has traveled far from their homeland to settle in new places, instigating multiple adjustment complexities [5]. The particular stressors of war play a large role in their struggles, but additionally, this population of refugees often face challenges typical to new migrants such as marginalization, acculturation problems, socioeconomic disadvantages and ‘cultural bereavement’ [6, 7], which all negatively impact their overall wellbeing. There are numerous practical obstacles to maintaining good health as well, such as a lack of transportation, choice of health institutions, poor nutrition, unstable and unsafe living conditions, language problems, lack of education, economic inadequacy, and a lack of social security [8].

Numerous studies have been conducted on various healthcare outcomes in the refugee population, yielding a traditional health response focused on the provision of services that predominantly address communicable diseases [9]. Other dominant areas of medical services distribution included treating cardiovascular diseases, hypertension or diseases with severe and immediate consequences owing to an interruption in management such as provision of insulin for type 1 diabetics or dialysis for kidney disease [10, 11]. Cancer care, unfortunately, remains an acutely neglected part of the refugee health response and is an under-analyzed issue as only a limited number of studies address this subject. In this study, we have set out to evaluate the rate of radiation therapy utilization among Syrian refugees with cancer living in Turkey and identify the contributing factors predictive of non-compliance with prescribed radiation therapy (RT).

## Methods

We performed a retrospective review of 14 Turkish institutional databases to identify Syrian refugee patients in Turkey with a cancer diagnosis from January 2015 to December 2019. Institutions were selected based on location within provincial regions known to host the highest numbers of Syrian refugees per United Nations High Commissioner for Refugees data [12]. All institutions were members of the Turkish Radiation Oncology Society (Türk Radyasyon Onkolojisi Derneği). We then identified those patients treated with RT and surveyed the demographic data, toxicity outcomes, and treatment compliance rates. We included patients who underwent external beam RT and/or brachytherapy. Patients who were treated with palliative intent were also included. Patients younger than 18 years old were excluded. Early toxicity outcomes were graded by the treating physician during the treatment course using the National Cancer Institute Common Terminology Criteria for Adverse Events (CTCAE), version 3.0.

Patient compliance with prescribed radiation treatments was scored as a binary variable. All radiation treatments were once-daily. Patients who missed 2 or more scheduled RT appointments were deemed “noncompliant.” Scheduled treatments that were cancelled because of issues related to the machine or physician-ordered planned treatment breaks did not count toward patient noncompliance. Follow-up was defined as the period from the time of completion of radiation therapy. Multiple variables that were tested as predictors of noncompliance included patient age, sex, diagnosis, residence of the patients, history of surgery, receipt of concurrent chemoradiotherapy, treatment length, and an acute toxicity of Grade ≥ 3. Pearson’s chi-squared test was used to determine the statistically significant differences in patients’ subgroups defined by these variables. Univariate and multivariate logistic regression analyses were also performed to test for an association between patient noncompliance and the variables listed above.

Statistical analyses were performed using SPSS statistical software version 25 (IBM Corp., Armonk, NY, USA). A *p*-value less than 0.05 was considered statistically significant. This study was approved by the Institutional Review Board of Marmara University Pendik Research and Education Hospital (IRB 09.2019.615) and of every participating center.

## Results

We identified 10,537 refugee patients who were diagnosed with cancer during the study period. Refugee status was verified by the national refugee billing ID code associated with each patient’s treatment. A total of 1010 (9.6%) of these patients were treated with RT. Patient characteristics are summarized in Table 1. Median age at the time of RT initiation was 49 (range, 19–94), and 53% of patients were female. The most common diagnoses were breast cancer (30%) and lung cancer (14%). The majority of patients were diagnosed at an advanced stage (68% Stage III-IV). Forty-four percent of patients were living in a refugee camp and 56% in a house. Grade ≥ 3 acute toxicity was reported in 14% of the patients. Median follow-up was 6 months (range, 0–46 months).

**Table 1:** Characteristics of the patients included in this study

Age	49 (19–94)
Median (range)	N (%)
Gender	
Male	479 (47)
Female	531 (53)
History of Smoking	364 (36)
Family history of cancer	85 (8)
Residence	
Refugee Camp	442 (44)
House	568 (56)
Diagnosis	
Breast cancer	303 (30)
Lung cancer	137 (14)
CNS cancer	128 (13)
Head and neck cancer	110 (11)
GI cancer	101 (10)
Prostate cancer	69 (7)
Gyn cancer	64 (6)
Others	98 (9)
Stage	
I-II	202 (20)
III-IV	687 (68)
Concurrent chemoradiotherapy	264 (26)
Noncompliance	205 (20)
Grade 3–4 acute toxicity	139 (14)
Radiation therapy fractions	
Mean (SD)	19.8 (9.5)
Follow-up (mo)	
Median (range)	6 (0–46)
Values are number (percentage) unless otherwise noted	

In total, 20% of patients were deemed noncompliant based on the definition of two or more missed RT treatments. Rates of noncompliance in patient subgroups are shown in Table 2. There was no significant difference in RT course length in noncompliant patients compared to those who were compliant (mean number of RT fractions, 19.7 vs 19.8, *P* = 0.933). All treatments were completed on an outpatient basis.

**Table 2:** Noncompliance rates in patient subgroups

Characteristic	Noncompliance rate, n (%)	P
Sex		0.903
Male	98/479 (21)	
Female	107/531 (20)	
History of Smoking	73/364 (20)	0.886
Family history of cancer	10/85 (12)	0.041
Residence		<.001
Refugee Camp	146/442 (33)	
House	59/568 (10)	
Diagnosis		0.141
Breast cancer	65/303 (22)	
Lung cancer	24/137 (18)	
CNS cancer	25/128 (20)	
Head and neck cancer	25/110 (23)	
GI cancer	15/101 (15)	
Prostate cancer	16/69 (23)	
Gyn cancer	7/64 (11)	
Others	28/98 (28)	
Stage		0.192
I-II	46/202 (23)	
III-IV	128/687 (19)	
Concurrent chemoradiotherapy	62/264 (24)	0.134
Grade 3–4 acute toxicity	20/139 (14)	0.062

Bivariate and multivariable logistic regression results for associations with patient noncompliance are summarized in Table 3. Treatment with concurrent chemoradiotherapy was associated with noncompliance on multivariate analysis (odds ratio [OR] 1.61, 95% confidence interval [CI] 1.06–2.46, *P* = 0.023). Living in a refugee camp was also associated with increased noncompliance (OR 3.62, 95% CI 2.43–5.19, *P* < .001). Treatment length was not associated with RT compliance.

**Table 3:** Bivariate and multivariate logistic regression results for associations with patient noncompliance

	Univariate models		Multivariate model	
Characteristic	Unadjusted OR (95% CI)	P	Adjusted OR (95% CI)	P
Age	1.02 (0.92–1.01)	0.703	1.00 (0.99–1.01)	0.988
Sex				
Male	Reference		Reference	
Female	0.98 (0.72–1.33)	0.903	1.11 (0.78–1.59)	0.553
Residence				
House	Reference		Reference	
Refugee camp	4.26 (3.05–5.95)	<.001	3.62 (2.53–5.19)	<.001
Stage				
I-II	Reference		Reference	
III-IV	0.78 (0.53–1.14)	0.193	0.76 (0.50–1.13)	0.18
Concurrent chemoradiotherapy				
No	Reference		Reference	
Yes	1.29 (0.92–1.82)	0.135	1.61 (1.06–2.46)	0.023
Acute toxicity				
Grade ≤ 2	Reference		Reference	
Grade ≥ 3	0.62 (0.38–1.03)	0.064	0.79 (0.45–1.37)	0.399
Radiation therapy fractions	1.00 (0.96–1.02)	0.933	0.99 (0.72–1.01)	0.467

## Discussion

Radiotherapy is an evolving yet inseparable element in the realm of cancer management. It has a myriad of applications which include being a definitive curative option, in slowing cancer growth or as a palliative modality. Several studies have emphasized this importance of RT, showing that on an average more than half of all cancer patients require RT administration at least once during the course of their treatment [13, 14]. In view of this, every patient should be able to have access to this beneficial modality of treatment. However, as we review data from around the world, there is a glaring disparity in the availability of RT services, which contrasts significantly between high-income countries when compared to middle to low-income countries. To further broadly analyze this area of concern, a study done in 2013 demonstrated high income countries such as the United States to have an estimate of 5075 RT departments with a total of 8911 megavoltage machines. This significantly differs from the middle to low income countries which range between having from 590 to as low as 40 RT departments with only 60–1000 machines [15].

Studies focusing on the availability of RT in Europe have demonstrated that almost three out of four cancer patients who have an evidence-based indication for RT have access to it [16, 17]. In a stark contrast to this, low income countries in sub-Saharan Africa have an almost-complete absence of RT facilities [18, 19]. To specifically focus on the situation of RT in Turkey, in 2012 there were 95 radiotherapy centers with 201 megavoltage equipment treating 160,808 patients with expected 44% unmet need [20]. Even though there has been a definite increase in the development in the field of RT in Turkey over the past few decades, these resources are not sufficient to meet the demands of the ever-increasing burden of patients diagnosed with cancer each year who require these facilities [21, 22].

There are a number of possible reasons as to why we observe this disproportionate chasm between the high-income and low-income countries. To establish RT centers equipped with megavoltage machines, the country’s income classification plays a major role. A majority of the lower income countries still to this day struggle to provide RT services as they deal with the crippling issues of poverty and political instability, pushing services like radiation further down the list of things deemed crucial [23, 24]. Aside from this, other factors such as the availability of electricity and the presence of highly qualified trained personnel to oversee the particulars of treatment delivery also play a crucial part in contributing to the underservices of RT [15].

Despite these impediments, Turkey has not only provided refuge to those fleeing the Syrian turmoil but has made all aspects of the healthcare system accessible to those in need, free of cost. On further introspection into the situation of Syrian refugees and the utilization of RT, there is another important aspect that should not be overlooked. In this analysis we found that even though only a small proportion of Syrian refugee patients with cancer received RT due to the reasons we discussed above, a significant number of these patients were non-compliant with multiple scheduled RT sessions. Patient noncompliance is a major setback which eventually hinders the possibility of achieving cancer remission, increasing patient morbidity and mortality. Certain studies have shown that the chances of increase of local recurrence with treatment interruption is almost 2% per day [25]. Tumor repopulation, which is an accelerated growth in malignant tissue after treatment with radiation has been initiated, has been deemed as an integral factor in causing this cancer remission [26]. In a retrospective analysis done to ascertain the survival in patients with head and neck carcinoma, any interruption in treatment for more than two weeks was found to be a strong, statistically significant determinant of tumor persistence [27]. Apart from its obvious effects on cancer treatment, non-compliance also leads to other complications. In a study conducted in the United States, a ripple-like effect was observed, in which non-compliant patients, by missing their scheduled treatments, indirectly caused delay in treatments of other patients by occupying slots on linear accelerator machines. It was also noted that non-compliance ultimately led to a failure to adhere to follow-up evaluations and other interventions crucial to cancer management [28].

The noncompliance rates vary globally owing to various factors influencing treatment adherence across different nations. In a New York-based clinical investigation involving 1227 patients with various malignancies, Ohri et al concluded that around 20% of the study patients were non-compliant [29]. In another study conducted in a tertiary care center in India, Gupta et al demonstrated a non-compliance rate of almost 13% in 203 cancer patients [30]. As per our analysis of the 1010 refugee cancer patients in Turkey that were able to get treated with RT, almost 20% of them were non-compliant.

There are several factors which can eventually result in non-compliance to radiotherapy and these have been extensively researched in various studies. Significant correlations between non-compliance and low socioeconomic status (SES) have been identified by multiple researchers, with patients belonging to a lower SES group having higher rates of non-compliance [31, 32]. Another important predictor that has been reviewed is the duration of treatment course. Longer treatment courses have been linked to a greater percentage of patients with non -adherence [33]. In rural and suburban populations, the distance required to travel to avail RT services has also been implicated in leading to non-compliance. Patients living farther away from the treatment centers were shown to be more likely to forego RT [34, 35]. Older age groups have been associated with increased rates of non-compliance. Sharma et al. demonstrated in their study of 47 elderly patients with head and neck cancer, that nearly one-third of the study group were not compliant with treatment [36].

As being refugees in an unfamiliar land places them in dire and blindsiding circumstances, they are posed with an additional set of elements that lead to non-compliance when compared with the general population. We have set out to analyze these factors in further detail. Language and cultural barriers play a major role as this hinders communication between refugees and the native healthcare workers [37]. Refugees are also faced with competing priorities that include availing basic accommodation, security, hygiene, nutrition and education which take precedence over their need for cancer treatment [38]. Non-compliance factors that were extracted specifically in our study were concurrent chemoradiation and living in refugee camps. Some refugees who are financially capable, seek to find their own homes to rent, but many who do not have this luxury are provided temporary refugee camps to stay in on arrival. Patients who have been living in refugee camps when compared to homes are faced with a harsher living environment, contributing to their non-compliance to RT.

Turkey has stood as a beacon of hope to millions of refugees by aiding them in not only providing a home away from their homeland in times of need, but also in allowing these new immigrants access to the same basic human rights as their native citizens. The Turkish government has taken multiple steps to overcome the challenges faced by the Syrian refugees in making use of its healthcare facilities. Provision of translators to overcome the language barriers and appointment of sufficient qualified medical professionals has helped refugees immensely. Above all, healthcare is also provided free of cost, whether it is at the primary, secondary or tertiary levels, with means to alleviate their medical predicaments and ease their transition into the new society [39]. Although Turkey has played a crucial role in aiding those escaping the Syrian war, there is still a disparity between what refugees need and what they are granted. A multidisciplinary plan can be taken to address these affairs but, as the well-being of refugees is an international affair, it needs to have a global amalgamation of efforts to produce visible results. Organizations such as the United Nations (UN), the European Union (EU) and World Health Organization (WHO) can align their efforts with an even greater focus to attempt to alleviate this situation [40]. Effective implementation of services should also be ensured with an emphasis on accountability. Measures on a smaller scale can be taken by providing refugees with local working opportunities along with taking the time to teaching them the local dialect, to help them ease integration into the new society. Further studies also need to be done to fathom the finer nuances that refugees deal with on a daily basis as this information can prove invaluable in providing a possible solution to this complex situation. It is imperative that borders do not become barriers in providing optimal radiotherapy for these inconvenienced individuals, regardless of the geopolitical reality.

## Conclusions

Syrian refugees in Turkey with cancer have low rates of radiation treatment utilization and low treatment compliance rates. Residence in a refugee camp and treatment with concurrent chemoRT were associated with lower compliance. Further studies and interventions are needed to understand the causes of refugee patient treatment noncompliance and improve radiation utilization and treatment completion rates.

## Data Availability

All data analyzed during this study are available from the corresponding author on reasonable request.

## Abbreviations

CI: Confidence Interval
CTCAE: Common Terminology Criteria for Adverse Events
EU: European Union
OR: Odds Ratio
RT: Radiation therapy
SES: Socioeconomic Status
UN: United Nations
WHO: World Health Organization
